# Acute/subacute cerebral infarction (ASCI) in HIV-negative adults with cryptococcal meningoencephalitis (CM): a MRI-based follow-up study and a clinical comparison to HIV-negative CM adults without ASCI

**DOI:** 10.1186/1471-2377-11-12

**Published:** 2011-01-26

**Authors:** Shu-Fang Chen, Cheng-Hsien Lu, Chun-Chung Lui, Chi-Ren Huang, Yao-Chung Chuang, Teng-Yeow Tan, Nai-Wen Tsai, Chiung-Chih Chang, Wan-Chen Tsai, Wen-Neng Chang

**Affiliations:** 1 Department of Neurology, Chang Gung Memorial Hospital-Kaohsiung Medical Center, Chang Gung University College of Medicine, 123, Ta-Pei Road, Niaosung, Kaohsiung 833, Taiwan; 2 Department of Radiology, Chang Gung Memorial Hospital-Kaohsiung Medical Center, Chang Gung University College of Medicine, 123, Ta-Pei Road, Niaosung, Kaohsiung 833, Taiwan

## Abstract

**Background:**

Acute/subacute cerebral infarction (ASCI) in HIV-negative cryptococcal meningoencephalitis (CM) adults has rarely been examined by a series of MRI-based follow-up study. We studied a series of MRI follow-up study of CM adults and compared the clinical characters of those with ASCI and those without ASCI.

**Methods:**

The clinical characteristics and a series of brain MRI findings of seven CM adults with ASCI were enrolled for analysis. The clinical characteristics of another 30 HIV-negative CM adults who did not have ASCI were also included for a comparative analysis.

**Results:**

The seven HIV-negative CM adults with ASCI were four men and three women, aged 46-78 years. Lacunar infarction was the type of ASCI, and 86% (6/7) of the ACSI were multiple infarctions distributed in both the anterior and posterior cerebrovascular territories. The seven CM patients with ASCI were significantly older and had a higher rate of DM and previous stroke than the other 30 CM adults without ASCI. They also had a higher incidence of consciousness disturbance at presentation and had a poor prognosis.

**Conclusion:**

ASCI was found in 18.9% (7/37) of HIV-negative CM adults. Serial MRI follow-up studies may allow a better delineation of ASCI in this specific group of infectious disease and multiple lacunar infarctions was the most common type. Older in age and presence of DM and previous stroke were the significant underlying conditions. CM patients with ASCI also had a poor therapeutic outcome.

## Background

Cryptococcal meningoencephalitis (CM) caused by *Cryptococcus neoformans *infection is a serious central nervous system (CNS) infection in immunocompromised patients [1]. Among the immunocompromised states, human immunodeficiency virus (HIV) infection is an important one [1], but CM also occurs in individuals with an apparently immunocompetent state [2-5]. Disturbed cerebral blood flow in the early stages of CM has been noted [6], and the occurrence of cerebral infarct may influence the therapeutic outcome [6,7]. There are case reports of cerebral infarction in CM [8-12], but cranial magnetic resonance imaging (MRI)-based assessment has been rarely examined solely. Cerebrovascular diseases are also important neurologic complications in HIV-infected patients [8,13], therefore, the HIV-positive status may interfere with the interpretation of the impact of CM on cerebrovascular disease. For this consideration, we conducted an MRI-based study to assess the clinical and neuroimaging features of seven adult HIV-negative CM patients who had an acute/subacute cerebral infarction (ASCI) in the early stage of CM. For a clinical comparison, the clinical characteristics of another 30 HIV-negative adults with CM but without ASCI were also analyzed.

## Methods

Over a period of seven years (2001-2007), 54 adult patients aged >17 years at the Chang Gung Memorial Hospital (CGMH)-Kaohsiung were retrospectively identified as having CM. Of these 54 CM adults, 37 had been examined by brain MRI at admission. Of the 37 patients, seven were found to have ASCI. In the present study, the clinical presentations, laboratory data, and MRI findings of these seven patients were analyzed. The clinical data of the other 30 CM adults who also underwent brain MRI at admission but did not have ASCI were included for a comparative analysis. This retrospective study was approved by the ethics committee of Chang Gung Memorial Hospital (IRB 97-0467B).

In this study, CM was defined as in either of the following ways: (1) isolation of *C. neoformans *in one or more cerebrospinal fluid (CSF) cultures, positive CSF cryptococcal antigen (Ag) titer, or positive CSF India ink staining and clinical features of meningitis; or (2) isolation of *C. neoformans *in blood culture with clinical presentations of meningitis and typical CSF features [5]. Cerebral ischemic stroke was defined according to the WHO criteria [14], and cerebral infarction complicating CM was defined as ASCI demonstrated by brain MRI at admission for CM management. The MRI findings of ASCI were defined as follows [15,16]: lesions with a hyperintensity on diffusion-weighted images (DWIs) and a hypointensity on apparent diffusion coefficient (ADC) maps. Cerebral infarcts were classified as follows: lacunar infarction vs. large infarction, anterior (territories of anterior or middle cerebral arteries) vs. posterior (territories of vertebral, basilar, or posterior cerebral arteries) and single vs. multiple infarctions (at least 2 regions of infarction observed). Lacunar infarction (occlusion of deep perforating arterioles) was defined as a subcortical, cerebellar, or brain stem infarction with a diameter of <15 mm, and a large infarction (occlusion of large intracranial arteries) was defined as a lesion of diameter >15 mm. Brain MRI studies were performed on a 1.5T scanner (Sigma Horizon; GE Medical Systems; Milwaukee, IL, USA) and included axial and sagittal T1- and T2-weighted images. Gadolinium-DTPA was administered to all the patients before the coronal and axial T1-weighted images were captured. DWI (b-value = 1.000 s/mm^2^) and ADC map capturing were similarly performed. The methods involving the MR angiography (MRA) technique in our study included 3-D TOF of intracranial vessels and contrast MRA of neck vessels. Maximum intensity projection (MIP) images were reviewed on picture archiving and communication system (PACS). MIP MRA images were evaluated for stenosis, and the vascular distribution of lesions was demonstrated [17]. In the meanwhile, all 37 CM patients with ASCI received a series of chest x-ray and/or chest computed tomography (CT) study to examine the presence of cryptococcoma or not. In the study period, patients with CM were treated with given antifungal drugs including amphotericin B ± fluconazole. The therapeutic results of the included 37 CM pathients were evaluated by using Glasgow Outcome Scale [18]: score 1 = death, score 2 = persistent vegetative state, score 3 = severe disability, score 4 = moderate disability, score 5 = good recovery. The patients with a score of 1-3 were classified as the group of poor prognosis, while those with a score of 4 and 5, good prognosis.

For comparative analysis of the clinical and laboratory characteristics of the CM patients with and without ASCI, two separate statistical analyses were performed. First, the age; CSF lactate, total protein, and glucose levels; and WBC counts at the time of admission were logarithmically transformed to improve normality and were compared between the CM patients with and without ASCI by using Student's *t*-test. Data on clinical manifestations were compared between the two patient groups by using the chi-square test or Fisher's exact test. Second, stepwise logistic regression was used to evaluate the relationship between significant univariables and the presence of ASCI, with adjustments made for potential confounding factors. Variables with a zero cell count in a 2 × 2 table were eliminated from the logistic analysis, and only variables with a strong association and statistical significance (*p *< 0.05) were included in the final model. The statistical tests were two-tailed and conducted using the Statistical Analysis System software package, version 13.0 (2002; SAS Institute; Cary, NC, USA).

## Results

**Figure 1:**
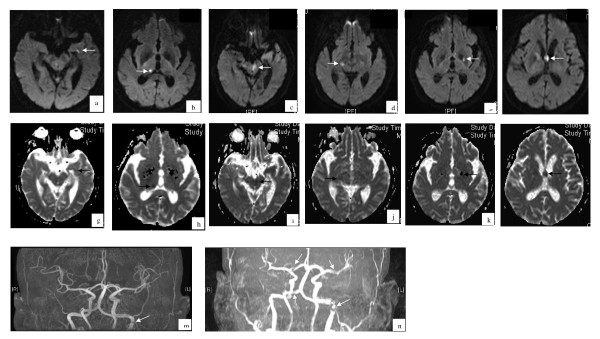
**Brain magnetic resonance imaging (MRI) (diffusion-weighted images (1a-1f) and corresponding apparent diffusion coefficient maps (1h-1m)) and MR angiographic (1g and 1n) studies of Case 5**. Figures 1a, 1b, 1h, and 1i (captured at the time of admission) show acute/subacute infarctions at the right thalamus (arrow) and left temporal region (arrowhead). Figure 1g (captured at the time of admission) shows suspected stenosis of the left internal carotid artery (arrow). Figures 1c-1f and 1j-1m (captured on the 17^th ^day after admission) show new infarctions in the midbrain (arrow), bilateral basal ganglia (arrowhead), and corpus callosum (arrows). Figure 1n (captured on the 17^th ^day after admission) shows multiple cerebral arterial stenoses (arrow).

The clinical characteristics and MRI findings of the seven enrolled patients are listed in Tables 1 and 2. The patients comprised four men and three women, aged 46-78 years (mean = 68.7 years). Underlying medical conditions were found in all seven patients, and all had altered consciousness as the clinical presentation at admission. The other clinical manifestations were fever in four patients, hemiparesis in four patients, seizure in one patient, and oculomotor palsy in one patient. The ASCI locations, MRA and other associated neuroimaging findings are presented in Table 1. Of the seven patients, Case 5 had recurrent ASCI in the early stage of CM, and his sequential MRI findings are shown in Figure 1. Pulmonary cryptococcoma was not found in the seven CM patients with ASCI, but it was found in two of the CM patients without ASCI.

**Table 1:** Basic clinical data, findings of magnetic resonance imaging, and therapeutic outcome of the 7 HIV-negative cryptococcal meningoencephalitis patients with acute/subacute cerebral infarctions

Case No	Age (y)/Sex	Clinical presentations (duration before admission)	Underlying conditions	Findings of magnetic resonance imaging			Outcome
					
				Sites of infarction	MRA	Others	
1	70/M	Headache, fever, altered consciousness, right hemiparesis (2 months)	Diabetes mellitus, chronic renal disease	Bilateral thalami, rostrum of corpus callosum	Mild narrowing of right MCA, right PCA, right ACA	-	Survived
2	73/M	Fever, altered consciousness, left hemiparesis (1 month)	Previous stroke	Pons: hemorrhage infarct, left midbrain, right posterior limb internal capsule	Narrowing of left VA	Leptomeningeal enhancement; ventricular dilatation, V-R space dilatation	Survived
3	78/M	Altered consciousness, fever, right hemiparesis, headache, seizure (2 weeks)	Hepatitis C	Splenium	Narrowing of right ACA, MCA, ICA	V-R space dilatation	Survived
4	46/F	Altered consciousness, fever (2 months)	Hepatitis B, liver cirrhosis, s/p liver transplantation	Right pons, right corona radiata, cerebellum, bilateral temporal region, bilateral basal ganglia, bilateral high frontal, right occipital	Narrowing of right VA	Leptomeningeal enhancement, ventricular dilatation	Died
**5**	75/M	Altered consciousness (1 week)	Diabetes mellitus	1^st^: Right thalamus, left temporal region; 2^nd^: bilateral basal ganglion, midbrain	Narrowing of left ICA, right ICA, left MCA, right VA	-	Survived
6	68/F	Fever, altered consciousness, headache (2 months)	Chronic renal disease, hepatitis C, liver cirrhosis, hepatocellular carcinoma, previous stroke	Midbrain, right temporoparietal region	-	Leptomeningeal enhancement, ventricular dilatation	Died
7	71/F	Fever, altered consciousness, right hemiparesis, oculomotor palsy (2 weeks)	Diabetes mellitus, hypertension	Midbrain, bilateral thalami	Poor opacification of bilateral PCAs	Leptomeningeal enhancement, ventricular dilatation, V-R space dilatation	Died

M = male; F = female; y = years; VR = Virchow-Robin; MCA = middle cerebral artery; ACA = anterior cerebral artery; ICA = internal carotid artery; PCA = posterior cerebral artery; VA = vertebral artery

**Table 2:** Comparison of cryptococcal meningoencephalitis patients with and without acute/subacute cerebral infarction

*Characteristics*	*with ASCI (n = 7)*	*without ASCI (n = 30)*	*p-value*
*Median age; range (years)*	*71 (46-78)*	*55 (19-82)*	0.033^*^
*Male*	*4*	*21*	*0.659*
*Female*	*3*	*9*	
*Underlying conditions*			
*Diabetes mellitus*	*3*	*2*	0.037^*^
*Liver disease*	*3*	*3*	*0.068*
*Hypertension*	*1*	*6*	*1.000*
*Clinical manifestation*			
*Fever*	*5*	*15*	*0.113*
*Altered consciousness*	*7*	*15*	0.028^*^
*Seizure*	*1*	*6*	*1.000*
*Previous stroke*	*2*	*0*	0.032^*^
*Hydrocephalus*	*4*	*11*	*0.408*
*Positive CSF India ink staining*	*0*	*10/27*	*0.145*
*Positive CSF culture*	*4*	*20/28*	*0.652*
CSF data median (range)*			
*WBC count (10*^*9*^*/L)*	*144 (33-160)*	*136 (0-980)*	*0.575*
*Glucose level (mmol/L)*	*26.5 (14-53)*	*24 (0-255)*	*0.827*
*Glucose ratio*	*0.16 (0.07-0.31)*	*0.19 (0-0.96)*	*0.731*
*Total protein level (g/L)*	*232.5 (113-431)*	*106 (55-1626)*	*0.131*
*Lactate level (mmol/dL)*	*44.95 (31-67)*	*47.6 (9-97)*	*0.687*
*Cryptococcus antigen*	*1:384 (1:4-1:1024)*	*1:1024 (1:4-1:4096)*	*0.265*
*Prognosis*			
*Poor*	*7*	*11*	*0.003*

CSF = cerebrospinal fluid; WBC = white blood cell; * = p < 0.05

The therapeutic outcomes of the seven CM patients with ASCI were that four patients (Cases 1, 2, 3, and 5) survived, while three (Cases 4, 6, and 7) died. Cases 1 and 3 were bedridden, and Cases 2 and 5 were wheelchair bound. All these seven patients belonged to the group of poor prognosis. As to the therapeutic results of the other 30 CM patients without ASCI, 19 had a good prognosis, while the other 11, a poor prognosis.

The comparative results of the seven patients with the other 30 patients without ASCI are listed in Table 2, and the significant findings were as follows: age at infection (*p = *0.033), altered consciousness on admission (*p = *0.028), diabetes mellitus (DM) (*p = *0.037), previous stroke (*p = *0.032), and poor prognosis (*p *= 0.003). The variables used in the logistic regression included age at infection and DM as the underlying conditions. After analysis, only DM was found to be independently associated with the presence of ASCI (*p *= 0.026, OR = 10.5, 95%, CI = 1.32-83.49).

## Discussion

The clinical presentations of CM are variable [1,19] and the pathophysiology of its neurologic sequelae is also complex [1,19]. Although many factors including positive cryptococcal antigenemia detection may influence the appearance and presentations of clinical CM [20,21], in patients with HIV-negative CM, little is known about the duration between the contact with *C. neoformans *and the occurrence of clinical symptoms as well as the duration between the initial onset of symptoms and the first consultation with a physician. In this study, all the seven CM patients with ASCI had altered consciousness and other focal neurologic signs for a varying period of time (1 week-2 months) before they came to our hospital for treatment. As shown in this study, the initial presentations of ASCI may be masked by or mixed with other clinical features, but they were detected by an early MRI study. CT study can show the various appearance of intracranial cryptococcosis [22], but the superiority of cranial MRI study to CT study in detecting the intracranial lesions of CM has been well demonstrated in the study of Charlier et al. [23]. Therefore, for thorough clinical evaluation, early MRI study using specific imaging sequences such as DWI and ADC maps should be mandatory for early detection of ASCI in HIV-negative CM patients.

It has been postulated that CM rarely results in cerebral infarction when compared with other fungal diseases [24,25] and that cerebral infarction is common in HIV-infected patients with CM [26,27], but these assumptions were not based on a thorough MRI study. Qureshi *et al. *[8] demonstrated an increased likelihood of cerebral infarction in HIV-infected patients compared with HIV-seronegative young individuals. Tjia *et al. *[28] found a 4% incidence of cerebral infarction secondary to CM in HIV-seronegative patients. In this MRI-based study, ASCI was found in 18.9% (7/37) of the HIV-negative CM patients.

We examined the risks factors of ASCI in HIV-negative CM patients, and the significant findings were as follows: First, altered consciousness on admission and DM and previous stroke as the underlying conditions are risk factors for ASCI. Second, a higher mean age at infection was a risk factor associated with cerebral infarction in HIV-negative CM patients. Therefore, in CM patients with the above-mentioned significant factors, the clinician should pay attention to the high possibility of ASIC occurrence in this group of patients.

With serial transcranial color-coded sonography (TCCS) and magnetic resonance angiography (MRA) studies, cerebral hemodynamic change was found to be last longer in CM than in acute bacterial meningitis [6,29], but a mismatch of the findings between TCCS and MRA studies in CM were also demonstrated [6]. Cerebrovascular complications are reported to be more common in chronic meningitis than in promptly treated acute symptomatic bacterial infections [29,30]. In the seven cases of this study, MRI detected ASCI within 1 week to 2 months of the development of clinical symptoms, and ASCI can recur during the therapeutic course, as shown in Case 5. This finding regarding the timing of the onset of ASCI is consistent with those reported in the case of chronic meningitis [12,31,32], which indicate that cerebral infarction can occur in both the acute and late stages during treatment for chronic meningitis.

There are several types of CM-related cerebrovascular events reported in the literature [8-12,25,33]. This study revealed that multiple cerebral infarctions were the most common type (86%; 6/7; Cases 1, 2, and 4-7)), and single infarction accounted for only 14% (1/7; Case 3). With regard to the distribution of the cerebral infarctions, both anterior and posterior cerebrovascular territories were involved in most patients. All the infarctions were lacunar, and no large cerebral infarction was noted. Hemorrhagic transformation was noted in only one patient (Case 2). In chronic meningitis, the inflammatory reaction is most intense in the basal meninges [6,12,34]. Because of this, basal exudates are usually most severe at the circle of Willis, and most cerebral infarcts are located in the territory of middle cerebral artery (MCA), particularly in the territories of the medial lenticulostriate and thalamoperforating arteries. In one murine model study of CM conducted by Charlier et al. [35], they showed that the primary event leading to CM was the parenchyma invasion after crossing the blood-brain-barrier at the cortical capillaries level surrounded by the Virchow-Robin space which may explain the high occurrence of brain lesions including ischemic events in this territory. However, actually, the stem and/or cortical branches of the MCA in the sylvian fissure, the supraclinoid portion of the internal carotid artery, and the vertebrobasilar system may also be involved [36-38]. This pathophysiological characteristic may explain why in our seven CM patients, ASCI was widely distributed, and almost all cerebrovascular territories were involved. Brainstem and/or cerebellum involvement were noted in 57% (4/7) of the HIV-negative CM adults with ASCI; this relatively high incidence of posterior fossa involvement in previously reported studies [8,9,12,13] were not MRI-based study. MRA for our seven CM cases showed varying degrees of arterial stenosis. Many mechanisms have been proposed for the cerebral vasculopathy in CM, including strangulation of vessels at the skull base, vasculitis, spasm, contraction, and thrombotic occlusion [6,9,39].

The therapeutic results of these seven patients showed a mortality rate of 43% (3/7), and all the survivors had serious neurologic deficits. This result is consistent with the previous belief that CM is a serious infectious disease of the CNS [1-3]. The CM patients with ASCI had a significant incidence of altered consciousness, as shown in Table 2, at the early stage of CM and had a poor prognosis of statistical significance when compared with those CM patients without ASCI. The prognostic influence of cerebral infarction on patients with chronic meningitis was also mentioned before [12].

## Conclusion

18.9% of adult HIV-negative CM patients have ASCI in the early stage of the disease. Multiple lacunar infarctions involving all cerebrovascular territories are common stroke features in this specific group of CNS infection, and most of the involved individuals are old and have other medical risk factors, such as DM and previous stroke, for ASCI. Our HIV-negative CM patients with ASCI also had a higher incidence of altered consciousness in the early stage of the disease and had a poor therapeutic outcome.

## Competing interests

The authors declare that they have no competing interests.

## Authors' contributions

All the authors read and approved the final version of this manuscript.

SFC had substantial contributions to conception and designed the study, carried out data acquisition and statistical analysis, drafted the manuscript and revised the manuscript. CHL had substantial contributions to design the study and carried out the clinical data analysis. CCL had carried out important neuroimaging acquisition and imaging analysis. CRH had substantial contributions to conception and carried out data analysis. YCC had substantial contributions to conception of the study and carried out clinical data analysis. TYT had substantial contributions to conception of the study and carried out data analysis, especially the stroke pattern analysis. NWT had substantial contributions to conception of the study and carried out statistical analysis. CCC had substantial contributions to conception of the study and carried out data analysis. WCT had substantial contributions to conception of the study and carried out data acquisition and analysis. WNC had substantial contributions to conception and designed the study, carried out data analysis, carried out critical revision and final approval of the revision.

## Pre-publication history

The pre-publication history for this paper can be accessed here:

http://www.biomedcentral.com/1471-2377/11/12/prepub
